# P-520. Clesrovimab Efficacy through 6 Months during a Time of Changing SARS-CoV-2 Nonpharmaceutical Interventions (NPIs): Subgroup Analysis of the China Cohort in the Phase 2b/3 CLEVER Trial

**DOI:** 10.1093/ofid/ofaf695.735

**Published:** 2026-01-11

**Authors:** Mingfen Zhu, Fang Sun, Rui Wang, Derong Liu, Chang Chen, Rong Fu, Jiahuang Lin, Yuanqiu Li, Qiong Shou, Andrea Guerra, Ying Zhang, Radha A Railkar, Anushua Sinha, Xueyan Liao

**Affiliations:** MSD, China, N/A, Beijing, China; MSD, China, N/A, Beijing, China; MSD, China, N/A, Beijing, China; MSD, China, N/A, Beijing, China; MSD, China, N/A, Beijing, China; MSD, China, N/A, Beijing, China; MSD, China, N/A, Beijing, China; MSD, China, N/A, Beijing, China; MSD, China, N/A, Beijing, China; MSD (UK) Limited, London, UK, London, England, United Kingdom; Merck & Co., Inc., Rahway, NJ, USA, Rahway, New Jersey; Merck & Co., Inc., Rahway, NJ, USA, Rahway, New Jersey; Merck & Co., Inc., Rahway, NJ, USA, Rahway, New Jersey; MSD, China, N/A, Beijing, China

## Abstract

**Background:**

Clesrovimab, an investigational long-acting monoclonal antibody, reduced the incidence of respiratory syncytial virus (RSV)–associated disease in healthy infants compared with placebo in the phase 2b/3 CLEVER trial (MK-1654-004; NCT04767373). The global trial was conducted during the COVID-19 pandemic when NPIs were widely implemented, disrupting RSV epidemiology and seasonality. In China, RSV cases were low from Jun 2022 to Feb 2023, a period overlapping with trial enrolment and follow-up, constraining the ability to demonstrate the protective effect of clesrovimab. This analysis evaluates the efficacy of clesrovimab within the context of implementation of NPIs in China during the CLEVER trial.

**Figure 1:**
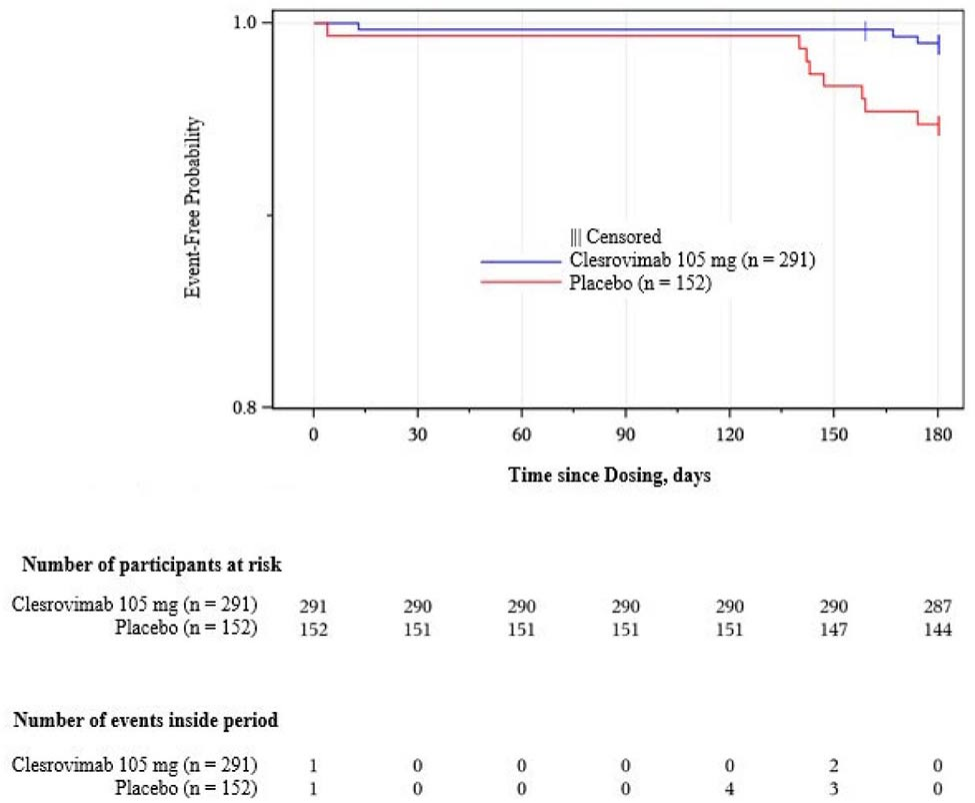
. Kaplan-Meier analysis of time and number of events from dosing to first RSV-associated outpatient or inpatient MALRI from Day 1 through Day 180 in China

**Methods:**

Healthy preterm and full-term infants ≤ 1 year old entering their first RSV season were randomized 2:1 to receive 1 intramuscular dose of clesrovimab 105 mg or placebo. RSV-associated medically attended lower respiratory infection (MALRI), requiring ≥ 1 indicator of lower respiratory infection/severity, was assessed through days 150 and 180 post dose during a period of changing SARS-CoV-2 NPIs. Analyses of the China subpopulation followed the overall study methodology of the CLEVER trial.

**Results:**

In China, enrolment spanned from Aug 20 to Dec 26, 2022, with the last Day 180 follow-up on June 24, 2023. Over 30 study sites, 443 participants received study intervention (clesrovimab, n = 291; placebo, n = 152). Participant disposition was generally comparable between intervention groups. Administration of clesrovimab reduced the incidence of RSV-associated MALRI (outpatient and inpatient) compared with placebo, with an observed efficacy of 89.6% (95% CI, 11.0%-98.8%) and 80.6% (95% CI, 27.3%-94.8%) through Days 150 and 180 post dose, respectively. RSV-associated MALRI cases were low or absent in both groups from Days 1-120 post dose (Dec 2022–Apr 2023) but began to increase following the easing of NPI, starting from Jan 2023, with a higher incidence observed in the placebo versus clesrovimab group from days 140-180 post dose (7 vs 2 cases; Figure 1).

**Conclusion:**

In China, clesrovimab provided sustained protection against RSV-associated MALRI beyond Day 150 through Day 180 post dose, particularly beyond Day 140, when the majority of cases began to emerge following the easing of NPIs.

**Disclosures:**

Mingfen Zhu, MSc, MSD, China: Employment Fang Sun, MD, Merck & Co., Inc., Rahway, NJ, USA: Stocks/Bonds (Public Company)|MSD, China: Employment Rui Wang, MSc, MSD, China: Employment Derong Liu, PhD, MSD, China: Employment Chang Chen, PhD, MSD, China: Employment Rong Fu, PhD, MSD, China: Employment Jiahuang Lin, PhD, MSD, China: Employment Yuanqiu Li, PhD, MSD, China: Employment Qiong Shou, PhD, MSD, China: Employment Andrea Guerra, MBBS, MSD (UK) Limited, London, UK: Employment Ying Zhang, PhD, Merck & Co., Inc., Rahway, NJ, USA: Employment|Merck & Co., Inc., Rahway, NJ, USA: Stocks/Bonds (Public Company) Radha A. Railkar, PhD, Merck & Co., Inc., Rahway, NJ, USA: Employment|Merck & Co., Inc., Rahway, NJ, USA: Stocks/Bonds (Public Company) Anushua Sinha, MD, Merck & Co., Inc., Rahway, NJ, USA: Employment|Merck & Co., Inc., Rahway, NJ, USA: Stocks/Bonds (Public Company) Xueyan Liao, PhD, Merck & Co., Inc., Rahway, NJ, USA: Stocks/Bonds (Public Company)|MSD, China: Employment

